# Infectious salmon anaemia virus (ISAV) isolated from the ISA disease outbreaks in Chile diverged from ISAV isolates from Norway around 1996 and was disseminated around 2005, based on surface glycoprotein gene sequences

**DOI:** 10.1186/1743-422X-6-88

**Published:** 2009-06-26

**Authors:** Frederick SB Kibenge, Marcos G Godoy, Yingwei Wang, Molly JT Kibenge, Valentina Gherardelli, Soledad Mansilla, Angelica Lisperger, Miguel Jarpa, Geraldine Larroquete, Fernando Avendaño, Marcela Lara, Alicia Gallardo

**Affiliations:** 1 Department of Pathology and Microbiology, OIE Reference Laboratory for ISA, Atlantic Veterinary College, University of Prince Edward Island, 550 University Ave., Charlottetown, P.E.I., C1A 4P3, Canada; 2 Biovac S.A., Bilbao 263, Puerto Montt, Chile; 3 Department of Computer Science and Information Technology, University of Prince Edward Island, 550 University Ave., Charlottetown, P.E.I., C1A 4P3, Canada; 4 Marine Harvest S.A., Puerto Montt, Chile; 5 National Fisheries Service (Sernapesca), Chile

## Abstract

**Background:**

Infectious salmon anaemia (ISA) virus (ISAV) is a pathogen of marine-farmed Atlantic salmon (*Salmo salar*); a disease first diagnosed in Norway in 1984. For over 25 years ISAV has caused major disease outbreaks in the Northern hemisphere, and remains an emerging fish pathogen because of the asymptomatic infections in marine wild fish and the potential for emergence of new epidemic strains. ISAV belongs to the family *Orthomyxoviridae*, together with influenza viruses but is sufficiently different to be assigned to its own genus, *Isavirus*. The *Isavirus *genome consists of eight single-stranded RNA species, and the virions have two surface glycoproteins; fusion (F) protein encoded on segment 5 and haemagglutinin-esterase (HE) protein encoded on segment 6. However, comparision between different ISAV isolates is complicated because there is presently no universally accepted nomenclature system for designation of genetic relatedness between ISAV isolates. The first outbreak of ISA in marine-farmed Atlantic salmon in the Southern hemisphere occurred in Chile starting in June 2007. In order to describe the molecular characteristics of the virus so as to understand its origins, how ISAV isolates are maintained and spread, and their virulence characteristics, we conducted a study where the viral sequences were directly amplified, cloned and sequenced from tissue samples collected from several ISA-affected fish on the different fish farms with confirmed or suspected ISA outbreaks in Chile. This paper describes the genetic characterization of a large number of ISAV strains associated with extensive outbreaks in Chile starting in June 2007, and their phylogenetic relationships with selected European and North American isolates that are representative of the genetic diversity of ISAV.

**Results:**

RT-PCR for ISAV F and HE glycoprotein genes was performed directly on tissue samples collected from ISA-affected fish on different farms among 14 fish companies in Chile during the ISA outbreaks that started in June 2007. The genes of the F and HE glycoproteins were cloned and sequenced for 51 and 78 new isolates, respectively. An extensive comparative analysis of ISAV F and HE sequence data, including reference isolates sampled from Norway, Faroe Islands, Scotland, USA, and Canada was performed. Based on phylogenetic analysis of concatenated ISAV F and HE genes of 103 individual isolates, the isolates from the ISA outbreaks in Chile grouped in their own cluster of 7 distinct strains within Genotype I (European genotype) of ISAV, with the closest relatedness to Norwegian ISAVs isolated in 1997. The phylogenetic software program, BACKTRACK, estimated the Chile isolates diverged from Norway isolates about 1996 and, therefore, had been present in Chile for some time before the recent outbreaks. Analysis of the deduced F protein sequence showed 43 of 51 Chile isolates with an 11-amino acid insert between ^265^N and ^266^Q, with 100% sequence identity with Genotype I ISAV RNA segment 2. Twenty four different HE-HPRs, including HPR0, were detected, with HPR7b making up 79.7%. This is considered a manifestation of ISAV quasispecies HE protein sequence diversity.

**Conclusion:**

Taken together, these findings suggest that the ISA outbreaks were caused by virus that was already present in Chile that mutated to new strains. This is the first comprehensive report tracing ISAV from Europe to South America.

## Background

Infectious salmon anaemia virus (ISAV) is a pathogen of marine-farmed Atlantic salmon (*Salmo salar*); a disease first diagnosed in Norway in 1984 [1]. For over 25 years ISAV has caused major disease outbreaks in the Northern hemisphere, and remains an emerging fish pathogen because of the asymptomatic infections in marine wild fish and the potential for emergence of new epidemic strains. ISAV belongs to the family *Orthomyxoviridae*, together with influenza viruses [2]. However, the virus is sufficiently different from influenza viruses to be assigned to its own genus, *Isavirus*. Sequence analysis of several ISAV isolates on the eight genomic segments consistently reveals two genotypes that are designated with respect to their geographic origin, European and North American; the two show 15–19% difference in their amino acid sequences of the fusion (F) and the haemagglutinin-esterase (HE) glycoproteins [3]. Since we now have ISAV isolates of both genotypes from Europe, North America, and South America, it has been proposed to drop the geographical designation of the genotypes and instead designate the European genotype as Genotype I and the North American genotype as Genotype II [4]. A sub-classification of the European (Genotype I) isolates into three clades (EU-G1, EU-G2, and EU-G3) has been proposed based on the 5' 1 kb of segment 6 sequences [5]. Additionally, results from phylogenetic analyses performed separately for each gene segment showed different phylogenetic relationships, with several European isolates diverging in virulence clustered together in several segments with high bootstrap support [6]. ISAV isolates can be further differentiated on the basis of insertion/deletions in a highly polymorphic region (HPR) spanning residues ^337^V to M^372 ^in the stem of the HE protein, adjacent to the transmembrane region: 26 different European and 2 North American HPR groups have been identified so far [7,8]. On the other hand, the HPR is vaguely defined, the HPR groups and numbering vary between publications or research labs; for example, Gagné and Ritchie [9] have suggested that the HPR should start from residue ^320^V/L since some isolates have deletions 5' of ^337^V. Moreover, use of HPR groups in epidemiological investigations was recently rejected because they vary significantly and are not suited as an indicator of relatedness between virus isolates [10]. Nonetheless, for both Genotypes I and II isolates, the HPR is an important virulence marker as a direct molecular relationship can be demonstrated between the HE protein stem length, ISAV cytopathogenicity in cell culture, and ability to cause clinical disease in Atlantic salmon [3,11]. The non-cultivable, non-pathogenic viruses detectable only by RT-PCR have the full-length HE protein (designated HPR0 or HPR00 for Genotype I found in Europe or North America, respectively) [7]. Because there is presently no universally accepted nomenclature system for designation of genetic relatedness between ISAV isolates, further investigations of different ISAV isolates from different geographical areas are necessary to facilitate comparison of ISAV isolates.

The first outbreak of ISA in marine-farmed Atlantic salmon in the Southern hemisphere occurred in Chile starting in June 2007 and has been reported [4]. In order to describe the molecular characteristics of the virus so as to understand its origins, how ISAV isolates are maintained and spread, and their virulence characteristics, we conducted a study where the viral sequences were directly amplified, cloned and sequenced from tissue samples collected from several ISA-affected fish on the different fish farms with confirmed or suspected ISA outbreaks in Chile. This paper describes the genetic characterization of a large number of ISAV strains associated with extensive outbreaks in Chile starting in June 2007, and their phylogenetic relationships with selected European and North American isolates that are representative of the genetic diversity of ISAV.

## Results and discussion

### RT-PCR, gene sequencing and analysis

**Figure 1:**
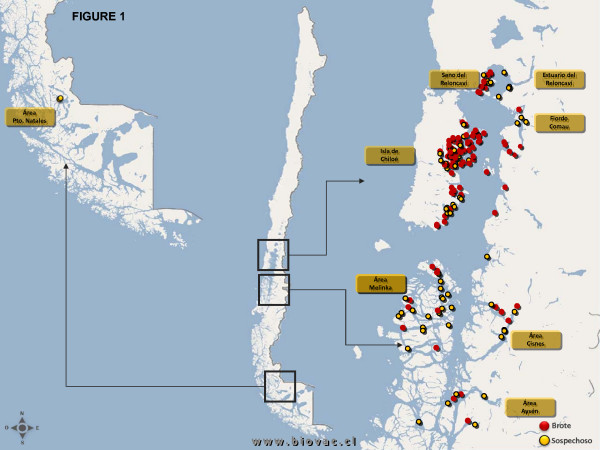
**Distribution of the ISA outbreaks in Chile; A map of Chile showing the location of the salmon aquaculture farms affected by the ISA outbreaks**. The three regions (represented by boxes) correspond to in Puerto Montt and Chiloé in Region X, Melenka and Aysén in Region XI and Pto. Natales in Region XII. Red denotes confirmed ISA outbreaks and yellow denotes suspected ISA outbreaks prior to laboratory confirmation.

**Figure 2:**
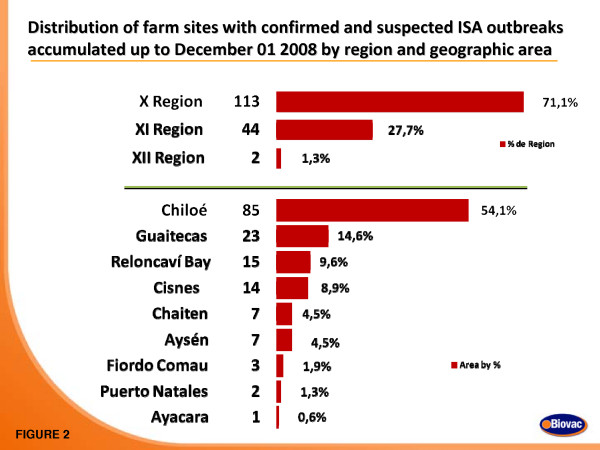
**Distribution of the ISA outbreaks in Chile; Chart showing the distribution of the accumulated total of salmon farms in Chile with ISA in Regions X, XI, and XII by December 01, 2008**.

As of November 2008, 159 accumulated total of salmon farms in Chile had been registered as positive for ISA by Sernapesca [12], of which 113 were in X Region, 44 in XI Region, and 2 in XII Region (Figures 1 and 2; [13]). From June 2007 to November 2008, a total of 242 tissue samples were collected from several ISA-affected fish on different fish farms with confirmed or suspected ISA outbreaks. In order to thoroughly describe the molecular characteristics of the viruses so as to understand their origins and virulence characteristics, we directly amplified viral sequences from the tissue samples by RT-PCR. It was considered that such a strategy would allow detection of the widest range of viral mutants associated with the ISA outbreaks. In the present study, the PCR products of the ISAV F and HE glycoprotein genes were cloned and sequenced or in some cases partial sequences were obtained by directly sequencing the PCR products without molecular cloning. All tissue samples received in viral transport medium were positive by RT-PCR for segments 5 and 6, and products containing full-length open reading frames (ORFs) were processed for cloning and sequencing. In contrast, some of the tissue samples received in RNAlater^® ^did not yield RT-PCR products for either RNA segment 5 or 6 full-length ORFs. In most of these cases, RT-PCR targeting the segment 6 HPR yielded a product, which was cloned and/or sequenced. We present here the sequence analysis of the ISAV envelope protein genes, F and HE, from several fish farms belonging to 14 different fish companies affected by the ISA outbreaks in Chile for 51 and 78 new isolates, respectively [see Additional file 1].

### Phylogenetic analysis of combined ISAV F and HE glycoprotein genes is a better approximation of genetic relatedness between ISAV isolates

**Figure 3:**
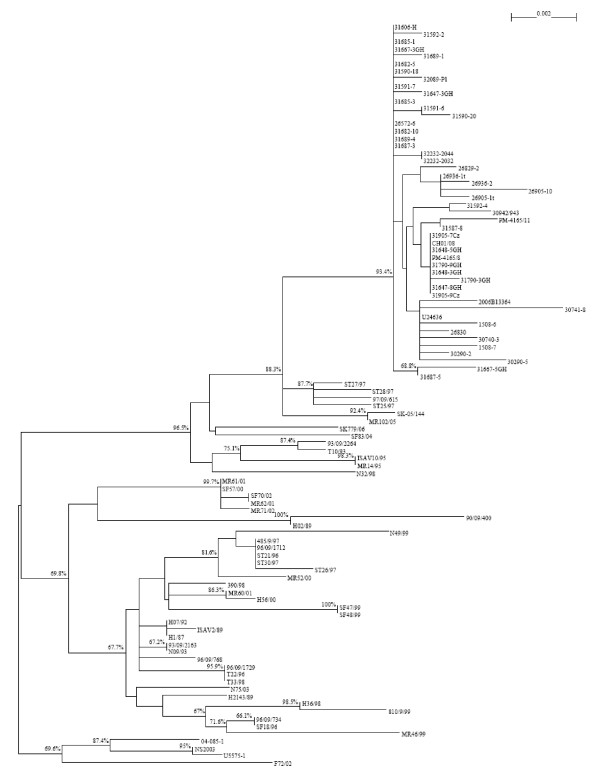
**Phylogenetic trees showing the relationships between the different ISAV isolates; RNA segment 5 showing the relationships between ISAV Genotype 1 isolates**. For easy identification of the phylogenetic trees, some branches have been marked with letters or numbers; a letter or a number represents all the isolates in that branch.

**Figure 4:**
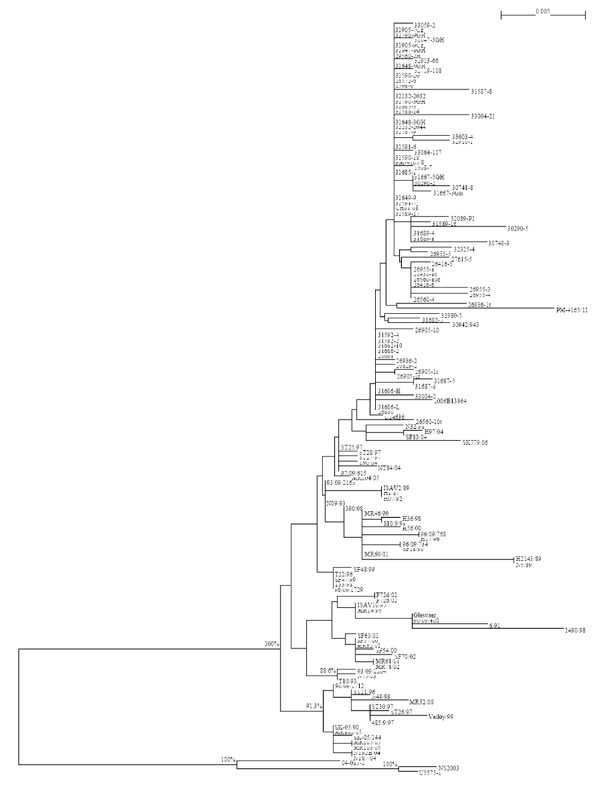
**Phylogenetic trees showing the relationships between the different ISAV isolates; RNA segment 6 showing the relationships between ISAV Genotype 1 isolates**. For easy identification of the phylogenetic trees, some branches have been marked with letters or numbers; a letter or a number represents all the isolates in that branch.

Phylogenetic analysis for segment 5 (F gene) of the new Chile ISAVs and other existing segment 5 sequences of reference isolates sampled from Norway, Faroe Islands, Scotland, USA, and Canada, 108 segment 5 sequences in total [see Additional file 1], was done. All ISAVs grouped into the two established genotypes, Genotype I (European) and Genotype II (North American), with the new Chile ISAVs isolated from the ISA outbreaks grouping within Genotype I [see Additional file 2]. To examine Genotype I more thoroughly, all ISAVs belonging to Genotype II were removed, and a tree was generated for Genotype I ISAVs only. This tree, which is shown in Figure 3, confirmed that the new Chile ISAVs are unique, grouping in their own cluster with considerable bootstrapping value (93.4%). These ISAVs are most closely related phylogenetically to the Norwegian ISAVs isolated in 1997 (ST28/97, ST25/97, ST27/97, and 97/09/615 (also referred to as ISAV8)) and 2005 (SK-05/144 and MR102/05). Bootstrap values indicate a more distant relationship to HPR0 viruses detected in Norway in 2004 (SF83/04) and 2006 (SK 779/06) [6]. A phylogenetic tree was generated with 156 segment 6 sequences [see Additional file 3]. Similarly to the segment 5 phylogenetic trees, all ISAVs examined also unequivocally grouped into the two established genotypes, Genotype I (European) and Genotype II (North American) on the segment 6 phylogenetic tree, with the new Chile ISAVs forming a unique cluster within Genotype I. Figure 4 shows the segment 6 tree for Genotype I ISAVs only, which clearly supports the two European genogroups, European-in-North America, and Real European with two clades inside the Real European genogroup, EU-G1 exclusive and EU-G2 with EU-G3, and all the new Chile ISAVs isolated from the outbreaks are in EU-G3. Thus whereas the boundaries between EU-G1, EU-G2 and EU-G3 were not clear in segment 5 (Figure 3; [see Additional file 2]), in segment 6, it is the boundary between the second EU-G2 and EU-G3 that are not clear (Figure 4; [see Additional file 3]), although the three clades EU-G1, EU-G2, and EU-G3 [5,8] are clearly recognizable within Genotype I (European Genotype). However, in the present trees (Figures 3 and 4) the European-in-North American ISAVs cluster separately from all EU-G2, showing them as a distinct genogroup within Genotype I.

**Figure 5:**
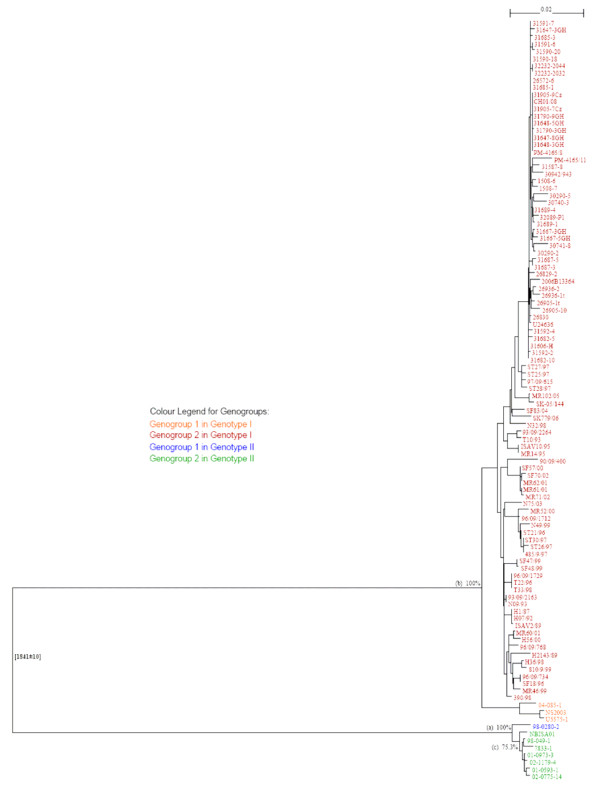
**Phylogenetic trees showing the relationships between the different ISAV isolates; Combined RNA segments 5 and 6 showing the relationships between all ISAV isolates**. For easy identification of the phylogenetic trees, some branches have been marked with letters or numbers; a letter or a number represents all the isolates in that branch.

**Figure 6:**
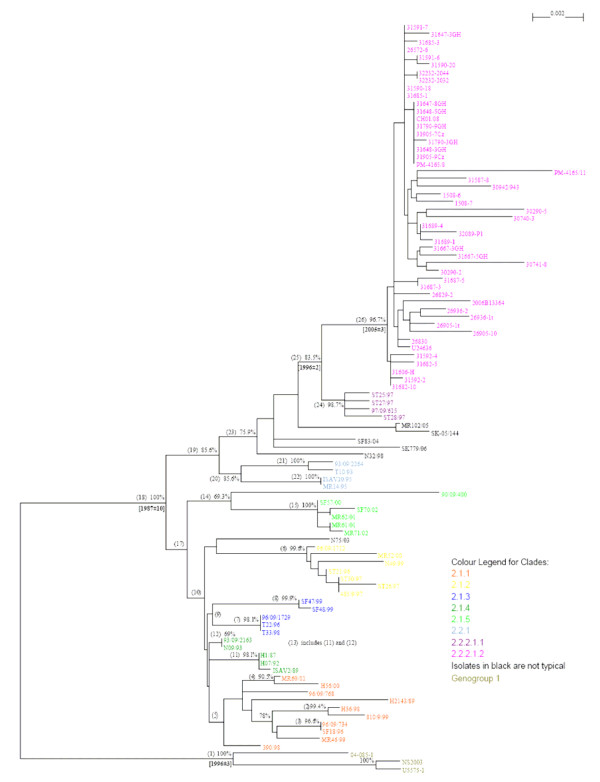
**Phylogenetic trees showing the relationships between the different ISAV isolates; Combined RNA segments 5 and 6 showing the relationships between ISAV Genotype I isolates**. For easy identification of the phylogenetic trees, some branches have been marked with letters or numbers; a letter or a number represents all the isolates in that branch.

**Figure 7:**
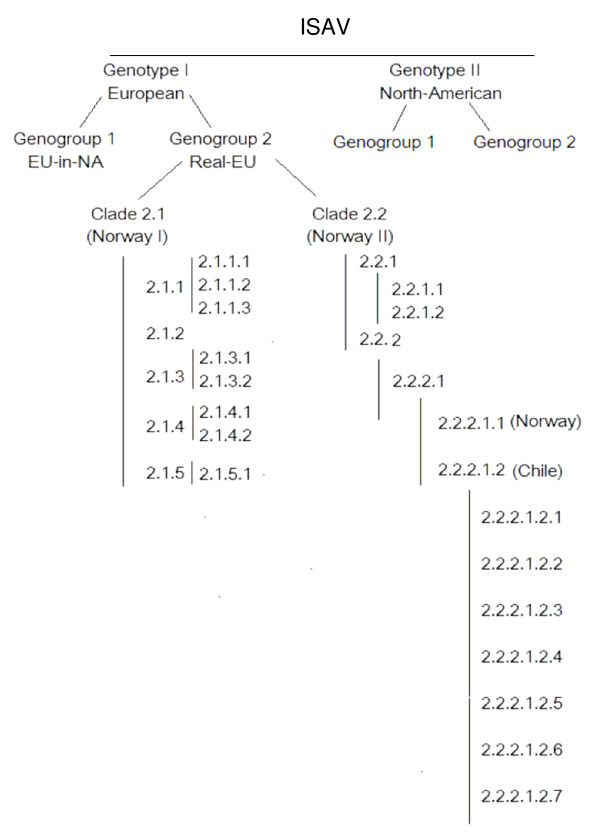
**Proposed nomenclature for ISAV species**.

It is apparent that the phylogenetic trees for ISAV segments 5 and 6 (Figures 3 and 4; [see Additional files 1 and 2]) are different, and it is not known which tree reflects the evolutionary history of the ISAV species. Since the complete genomic information of all the isolates is not available, it can be reasonably expected that a phylogenetic tree generated based on the combination of segments 5 and 6 will provide a better approximation of genetic relatedness between virus isolates than the tree based on either segment 5 or 6 alone, not withstanding the possibility that these genes evolve independently. The present study produced a new sequence by concatenating the segment 5 sequence and the segment 6 sequence for each isolate with the rationalization that these new sequences would approximate the real phylogenetic relationship more closely. Figure 5 shows the phylogenetic tree for 106 of the ISAV isolates for which both segments 5 and 6 sequences were analyzed [see Additional file 1], and Figure 6 shows the detailed tree for the Genotype I portion of this tree. High bootstrapping values (more than 65%) are marked in Figures 5 and 6. For easy identification of the phylogenetic trees, some branches have been marked with letters or numbers. To our knowledge, this kind of concatenation and tree generation has not been done before. It is proposed that this tree, which incorporates an arbitrary numbering system of two unique first-order clades each (clades 1 and 2) in Genotypes I and II, be the basis for the nomenclature and genotyping for ISAV, and a proposed uniform nomenclature for ISAV species is illustrated in Figure 7.

The evolution of Genotype II segments 5 and 6 genes, in contrast to Genotype I, is extremely limited, and the 8 reference ISAV isolates analyzed can only be grouped into two genogroups: Genogroup 1 consisting of isolate 98-0280-2, and Genogroup 2 containing the remaining ISAV isolates, but no higher-order clades beyond this grouping (Figure 5).

The phylogenetic tree for the 98 Genotype I ISAVs (Figure 6) clearly supports the classifications of the individual segments 5 and 6, but also provides a better approximation of genetic relatedness between virus isolates than either segment 5 or 6 alone (Figures 3 and 4; [see Additional files 1 and 2]). All isolates of Genotype I (Figure 5) can be classified into two genogroups: Genogroup 1 (European-in-North America) is branch (1) and Genogroup 2 (Real-European) is branch (18) (Figure 6). Members of Genogroup 1 in Genotype I correspond to EU-G2 of Nylund *et al*. [5] together with those of branches (14) and (20). Within Genogroup 2 of Genotype I are two second-order clades, branch (17) corresponding to Clade 2.1 (Norway I) and branch (19) corresponding to Clade 2.2 (Norway II). The bootstrapping support value for (19) is pretty high, but not for (17), although considering Figures 3 and 4, this classification is reliable. Inside branch (17) there are five groups or branches that could be the candidates for the first level clades under Clade 2.1 (branches (5), (6), (9), (13) and (14) corresponding to clades 2.1.1, 2.1.2, 2.1.3, 2.1.4, and 2.1.5), although some of them, for example branch (5), have bootstrapping value below 65%. However, the three branches under (5), which are very close, have high bootstrapping values: the support for (2) which is clade 2.1.1.1 is 99.4%; the support for (3) which is clade 2.1.1.2 is 96.6%; and the support for (4) which is clade 2.1.1.3 is 90.5%. Clade 2.1.4 has two second level clades: branches (11) and (12) corresponding to clades 2.1.4.1 and 2.1.4.2. Members of clades 2.1.1, 2.1.3, and 2.1.4 correspond to EU-G3 whereas members of clade 2.1.2 are a mixture of EU-G1 and EU-G2 of Nylund *et al*. [5].

Clade 2.2 (Norway II), i.e., branch (19) (Figure 6), has two branches, (20) and (23), corresponding to first level clades 2.2.1 and 2.2.2, respectively. Inside branch (20), branches (21) and (22) can be named clades 2.2.1.1 and 2.2.1.2, respectively. Inside branch (23), branch (25) is the only stable clade and can be named 2.2.2.1, which separates into two additional third-order clades (2.2.2.1.1 [Norway] which is branch (24), and 2.2.2.1.2 [Chile] which is branch (26). Thus all the ISAVs from the disease outbreaks in Chile are unique, grouping in their own cluster, clade 2.2.2.1.2, and are most closely related phylogenetically to the Norwegian ISAVs isolated in 1997 (ST28/97, ST25/97, ST27/97, and 97/09/615 (also referred to as ISAV8)) which make up clade 2.2.2.1.1.

### More detailed analysis of the new Chile ISAV isolates identifies 7 distinct strains

**Figure 8:**
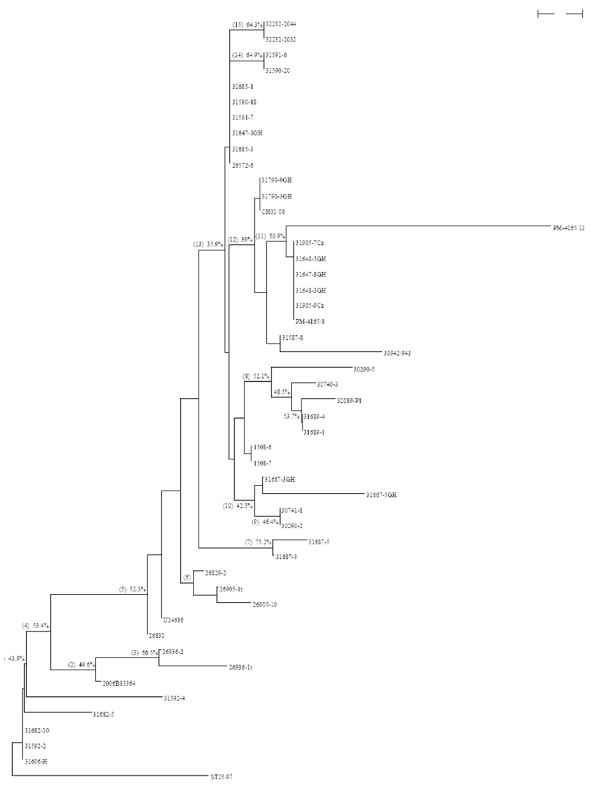
**Identification of ISAV strains among the new Chile ISAV isolates**. Since the sequences consisting of only informative columns are much shorter than the whole sequences, percentage distance is not meaningful anymore and therefore no scale legend is given. Branches have been marked with letters or numbers for easy identification in the tree.

Clade 2.2.2.1.2 consists of the ISAVs from the ISA outbreaks in Chile (48 isolates in total). To further explore the evolutionary relationships among these Chile isolates and find the possible stable clades inside this group, a fine phylogenetic analysis for these isolates was done. For this, the multiple alignment of the combined segments 5 and 6 sequences of the Chile isolates (48 isolates, maximal length 2,380 bp) were manually scanned; those columns that have single random mutations (mutations occuring in only one column and only in one sequence) or are identical for all isolates, and that have gaps due to length difference of sequences were deleted. Only those columns that have systematic mutations (mutations occuring in the same way in more than one sequence), that have continuous mutations (mutations occuring in continuous columns), and that have gaps due to evolutionary indel events were kept. These columns were called informative columns. A phylogenetic tree was then generated based on the informative columns of the combined segments 5 and 6 sequences of the new Chile isolates. Isolate ST25/97 was included in the tree as the outgroup. This tree is reported in Figure 8. Because the marginal gaps were manually removed, we could involve gaps in the bootstrapping process; the bootstrapping values that are higher than 35% are listed. Based on Figure 7, we can group all the Chile isolates into 7 different ISAV strains as also depicted in Figure 8 and Table 1. 

**Table 1:** New Chile ISAV Strains

**ISAV Clade**	**2.2.2.1.2.1**	**2.2.2.1.2.2**	**2.2.2.1.2.3**	**2.2.2.1.2.4**	**2.2.2.1.2.5**	**2.2.2.1.2.6**	**2.2.2.1.2.7**
	31682-10	31687-5	30290-5	31667-3GH	31648-5GH	32232-2044	26905-1t
	
	31592-2	31687-3	30740-3	31667-5GH	31647-8GH	32232-2032	26905-10
	
	31592-4		32089-P1	30741-8	31648-3GH	31591-6	26829-2
	
	31606H		31689-1	30290-2	PM-4165/8	31590-20	U24636
	
	31682-5		31689-4		31905-7cCz	31647-3GH	26830
	
	26936-2		1508-6		31905-9Cz	31685-1	
	
	26936-1t		1508-7		PM-4165/11	31685-3	
	
	2006B13364				31790-3GH	26572-6	
	
					31790-9GH	31591-7	
	
					CH01/08	31590-18	
	
					31587-8		
	
					30942/943		

The main characteristics and clinical history of the 7 different Chile ISAV strains are summarized [see Additional file 4]. The seven isolates belonging to Chile Strain 1 have no insert in segment 5, and belonged to multiple HPR groups on segment 6. Only three of the seven isolates were from confirmed ISA outbreaks. The other four Chile 1 isolates were from fish not diagnosed with ISA disease; one isolate was from Atlantic salmon parr, one isolate was from broodstock fish without any symptoms, and two isolates were from adult fish diagnosed with amoeba gill disease (*Neoparamoeba perurans*) [14]. It is possible that the amoeba disease was a concurrent infection with ISA. In contrast, Chile strains 2–7 were all from confirmed or suspected ISA outbreaks and all isolates had the 11-aa insert in segment 5 and their segment 6 sequences belonged to HPR 7b and/or HPR 7f except for Chile strain 7 which also had a mixed infection with HPR 2.

### Estimation of branching times of ISAV isolates shows new Chile ISAV strains diverged from Norway ISAV isolates around 1996

To establish the timing of the evolutionary process among ISAV isolates, we used the BACKTRACK program [3] to estimate the divergence time for some specific inner nodes of the phylogenetic trees shown in Figures 5 and 6. The mutation rates for ISAV segments 5 and 6 were previously determined as 0.67 × 10^-3 ^nucleotides per site per year and 1.13 × 10^-3 ^nucleotides per site per year, respectively [3]. Because Figures 5 and 6 were generated based on the combined segments 5 and 6 sequences for each isolate, the average mutation rate of 0.90 × 10^-3 ^nucleotides per site per year was taken as the mutation rate of the combined segments 5 and 6 sequences. The output of the BACKTRACK program is reflected in Figures 5 and 6 as the estimated divergence years shown in brackets. Thus within Genogroup 2 of Genotype I, Clade 2.1 (Norway I) Clade 2.2 (Norway II) diverged around 1987 with the interval of estimation of plus or minus 10 years. This event was probably associated with the first diagnosis of ISA in Norway in 1984. Within Clade 2.2 of Genogroup 2 in Genotype I, clade 2.2.2.1.2 [Chile] diverged from clade 2.2.2.1.1 [Norway] around 1996 with the interval of estimation of plus or minus 2 years. This timeline suggests that the ISA outbreaks in Chile were caused by virus that was already present in Chile that mutated to new strains. It has been suggested that the virus was introduced to Chile through fish egg imports from Norway in the past 10 years [15]. The analysis in the present study, which included 48 Chile ISAVs isolated between 2007 and 2008 gives a more specific introduction time of around 1996 (Figure 6). The long length of the branch under (26) in Figure 6 suggests that the introduced virus took a long time to evolve into the strains that caused the ISA outbreaks starting in June 2007. This would indicate that probably introduction occurred on a very small scale into one specific location following which a few years later the virus was disseminated into the Chilean Atlantic salmon industry. Most likely the introduction involved ISAV isolates of Chile Strain 1 (Clade 2.2.2.1.2.1, Figure 6, and Table 1 [see Additional file 4] or similar virus strain, and the wide dissemination in the industry occurred around 2005, two years before the first outbreak of ISA, which involved Chile Strain 7 (Clade 2.2.2.1.2.7), was recognized in marine-farmed Atlantic salmon in Chile [4].

### 11-amino acid insert in F protein unique to new Chile ISAV strains

Analysis of 51 virus isolates for which we had full-length ORF of the F gene showed 43 isolates with an 11-amino acid (aa) insert between ^265^N and ^266^Q, a mutation site previously postulated to be a marker for reduced virulence next to the putative proteolytic cleavage site ^267^RA/G^268 ^in the F protein [see Additional file 5]. The 11-aa insert has 100% sequence identity with RNA segment 2 of Genotype I, which encodes the PB1 polymerase. The mutations ^265^N → ^265^Y and ^266^L/Q/H → P^266 ^next to the putative proteolytic cleavage site ^267^RA/G^268 ^of the F protein are characteristic of ISAV of reduced pathogenicity [3]. Most recently, the F gene of HPR0, a non-pathogenic virus, was reported to have ^265^NQ^266 ^at this site, and it was proposed that the mutation ^266^Q → L^266 ^was a prerequisite for virulence, and that ISAV lacking this mutation required a sequence insertion near the cleavage site in order to gain virulence [6]. However, in the present study, all eight Chile isolates without the 11-aa insert [see Additional file 5] including the seven isolates in [see Additional file 4] identified to belong to Chile Strain 1 had the peptide ^265^NL^266 ^but only three isolates (26936, 2006B13364, and 31592), were not associated with confirmed ISA outbreaks. Before the Chile ISA outbreaks, there had been only 8 ISAV isolates with indels in RNA segment 5 [6,16]. All these isolates are found in Norway; seven of them were recovered between 1999 and 2002 [16] and one was recovered in 2006 [6]. Seven of these isolates had inserts from different parts of RNA segment 5 while in one isolate, the insert was shown to come from RNA segment 3, which encodes the ISAV nucleoprotein. As shown in [see Additional file 6], RNA segment 5 has an indel evolutional change involving 33 bp and can be classified into four cases: two major groups and two special cases. The bigger group includes most of the Chile isolates (43 isolates); the smaller group includes 6 Norway isolates (H2143/89, MR71/02, MR61/01, MR62/01, SF70/02, and SF57/00). The two special cases are Norway isolates MR60/01 and MR46/99. The significant point is that the indel sequences in the four cases are all different. The obvious difference among them indicates that this indel event should be an insertion instead of a deletion. We can therefore reasonably speculate that the original Chile ISAV sequences did not have this 33 bp sequence (11-amino acid sequence). Chile Strain 1 isolates [see Additional file 4] do not have this portion either. Later, insertion events occurred, likely by non-homologous recombination between the F and PB1 genes of the same virus resulting in Chile Strains 2 to 7. Based on the long branch under (5) in Figure 8, we may speculate that a small amount of Chile Strain 1 ISAV was introduced to a specific location inside Chile; it took a relatively long time (around two years) for a recombination event (insertion event) to happen, and then quickly disseminated to different locations and evolved to different strains. Insertions also occurred in Norway isolates, but the Norway insertion events were independent of the Chile insertion. At least three different Norway insertions occurred. Such mutations are well known in avian influenza virus (AIV), in case of recombination events involving a sequence insertion in the HA gene of AIV near the cleavage site of the protein associated with increased cleavage rate and leading to emergence of new virulent strains [17-20]. The presence of Chile Strain 1 ISAV was probably not detected for some time prior to the initial disease outbreak of June 2007 [4], which involved Chile Strain 7 [see Additional file 4].

### Multiple HPR groups in ISA outbreak is manifestation of ISAV quasispecies

Analysis of the deduced HE protein full-length sequence and HPR sequence revealed a total of 24 different HE-HPR groups, including HPR0, with HPR 7b making up 79.7% of the virus isolates analyzed in samples from different fish farms (Table 2; [see Additional file 7]. Other diagnostic labs in Chile found up to 9 different HE-HPR groups in the same outbreak (ISA workshop in Chile, Puerto Varas, November 19–20, 2007), including two recent separate detections of HPR0 virus, one of them at a site in an estuary in XII region. When all these HPR groups are considered together, there are at least 28 distinct HE-HPR variants associated with the ISA outbreaks in Chile to date. However, we do not consider this evidence of multiple co-circulating lineages of ISAV as the diverse 5' 1 kb HE sequences of the respective HPR groups appeared sequentially related to one another and with tight grouping by phylogenetic analysis, and were associated with only 7 distinct ISAV strains based on segments 5 and 6 phylogenetic analyses (Figure 8). The appearance of multiple HE-HPR groups in such a short time in tissues from the same or different fish originating from the same or different fish farms is considered to be a manifestation of ISAV quasispecies HE protein sequence diversity, and for the first time we can clearly show that the ISAV HPR groups exist as quasispecies populations ([see Additional file 7] although this will require confirmation by analysis of multiple clones derived from one clinical sample. Quasispecies viral populations are connected with a high potential for rapid evolution [21,22]. This high mutation rate of RNA viral replication is also suggested to be responsible for the non-homologous recombination between the ISAV F and PB1 genes in ISAV strains associated with the ISA outbreaks in Chile.

**Table 2:** Segment 6 highly polymorphic region (HPR) groups among the new Chile ISAV strains

**HPR group**	**No. of isolates sequenced^1^**	**Percent of total**
**HPR 7b**	192	79.7%

**HPR 7c**	1	0.4%

**HPR 7e**	1	0.4%

**HPR 7f**	3	1.2%

**HPR 7g**	1	0.4%

**HPR 7h**	1	0.4%

**HPR 7i**	2 (1 was mixed with HPR 7b)	0.8%

**HPR 1b**	1	0.4%

**HPR 1c**	7	2.9%

**HPR 2**	10	4.1%

**HPR 2c**	1 (mixed with HPR 2)	0.4%

**HPR 2d**	1	0.4%

**HPR 3**	6	2.5%

**HPR 3a**	1	0.4%

**HPR 4c**	3 (2 were mixed with HPR 7b)	1.2%

**HPR 5**	2	0.8%

**HPR 5c**	1	0.4%

**HPR 9b**	2	0.8%

**HPR 15**	1	0.4%

**HPR 15b**	1	0.4%

**HPR 15c**	1 (mixed with HPR 7b)	0.4%

**HPR 15d**	1	0.4%

**HPR 15e**	1	0.4%

**New HPR (12-aa deletion)**	5	2.1%

**HPR0**	1 (mixed with HPR 7b)	0.4%

^1^Total 241 isolates

Demonstration of the ISAV quasispecies HE protein sequence diversity in the present study was only possible by sequencing of RT-PCR products obtained directly from fish tissue. Such a strategy avoided the inadvertent strong selection that occurs during virus isolation/culture procedures using different fish cell lines, as well as the potential contamination with ISAV strains used in the laboratory, when viruses isolated in cell cultures are sequenced. Moreover, attempts to isolate virus from some natural ISA outbreaks and from some ISAV RT-PCR-positive fish are not always successful [23-28], and the virus isolates most probably do not reflect the spectrum of wild viruses and, therefore some local strains might have escaped surveillance if only cultivated virus isolates had been sequenced. HPR 7b was the most commonly detected HE-HPR group in tissue samples from different fish farms, accounting for 79.7% of the virus isolates analyzed. It is therefore reasonable to conclude that HPR 7b wild-type virus is the main cause of this outbreak [4].

It is remarkable that within a year of the Chile ISA outbreaks it was possible to detect HPR0 virus on three different occasions. ISAV HPR0 viruses are non-cultivable, are considered non-pathogenic [29,30], and are examples of frag-viruses [31] since they are known only through genomic sequence fragments. ISAV HPR0 viruses were first detected in wild Atlantic salmon in Scotland in 2002, four years after the ISA outbreak in UK [29], and in farmed Atlantic salmon in New Brunswick in 2004, eight years after the first ISA outbreak in Canada [30]. The rapid detection of HPR0 virus in association with the ISA outbreaks in Chile is most likely due to use of RT-PCR directly from fish tissue samples, with primers targeting ISAV RNA segments 6 and 8, and sequencing of the PCR products as the principal method of ISA laboratory diagnosis. In contrast, in both UK and Canada, ISAV RT-PCR was mostly used to confirm virus isolates from diseased fish and in most cases RT-PCR targeted ISAV RNA segment 8 only [23,32], which does not differentiate between HPR groups.

## Conclusion

In conclusion, 51 ISAV F and 78 HE sequences were directly amplified from tissue samples collected from several ISA-affected fish from June 2007 to November 2008 during the ISA outbreaks in Chile, and used to determine their phylogenetic relationships with selected European and North American isolates that are representative of the genetic diversity of ISAV. The phylogenetic tree based on combined sequences of segments 5 and 6 sequences for each isolate provided better understanding of the evolutionary relationship among ISAVs, showing that the new Chile isolates grouped in their own cluster of 7 distinct strains within Genotype I of ISAV. The phylogenetic software program, BACKTRACK, estimated the Chile isolates diverged from Norway isolates around 1996, and following a recombination event (insertion event) in the F gene around 2005, were quickly disseminated throughout the Atlantic salmon industry prior to the index case in June 2007. This is the first comprehensive report tracing ISAV from Europe to South America.

## Methods

### History of tissue samples collected from farmed Atlantic salmon in Chile

Moribund fish were submitted for laboratory analysis to the Biovac S.A. laboratory in Puerto Montt, Chile, where a full necropsy was conducted and tissue samples were collected for histological evaluation, virus isolation, and immunohistochemistry and molecular biology analysis. Figure 1 represents a map of the accumulated ISA outbreaks in Chile from June 2007 to November 2008. Original fish samples consisting of organ pools comprising liver, spleen, gill, heart and head kidney were received at the Atlantic Veterinary College (AVC), University of Prince Edward Island (UPEI), Canada, either in RNALater^® ^(Ambion Inc., Foster City CA) or in viral transport medium consisting of Hank's MEM with 10% FBS and 1% Antibiotic-Antimycotic (Invitrogen). Six additional samples of organ pools collected in 2008 in RNALater^® ^(Ambion Inc.) were received from two other private sources in Puerto Montt, Chile. In total, samples were obtained from 14 fish companies operating in Chile.

### RNA isolation, RT-PCR and gene sequencing

Total RNA was extracted from 375 μl volumes of tissue homogenates or cell culture lysates using TRIZOL LS Reagent (Invitrogen) prior to RT-PCR amplification. The RT-PCR amplification was performed with the Qiagen One Tube RT-PCR System kit (Qiagen) in a PTC-200 DNA Engine Peltier thermal cycler (MJ Research, Inc.) using oligonucleotide primers and cycling conditions as previously described [3,33]. In some cases RT-PCR targeting the RNA segment 6 HPR was performed. The HPR primers consisted of ISAV HPR Fwd 5'-GCC CAG ACA TTG ACT GGA GTA G-3', and ISAV HPR Rev 5'-AGA CAG GTT CGA TGG TGG AA-3'. The RT-PCR amplification conditions were 1 cycle at 50°C for 30 min, one cycle at 95°C for 15 min, 40 cycles at 94°C for 30 s, 60°C for 60 s and 72°C for 90 s and 1 cycle at 72°C for 10 min before soaking at 4°C. The PCR products were cloned into either the pCRII vector using a TOPO TA cloning kit (Invitrogen) or the pDrive Cloning Vector using the QIAGEN PCR cloning kit (Qiagen) in preparation for nucleotide sequencing, although in some cases the RT-PCR products were sequenced directly without cloning. Plasmid DNA for sequencing was prepared as described before [34], and DNA sequencing was performed as previously described [3]. Sequences are available through GenBank and their accession numbers are listed in [see Additional file 1].

### Phylogenetic analyses

A large set of ISAV isolates sequenced on either one or both RNA segments 5 and 6 was used. In order to guarantee the quality of the analyses, the existing sequences were obtained directly from GenBank [35], making sure they were unique, correct and current [see Additional file 1]. For each RNA segment, all the isolates were used in a multiple alignment by using CLUSTAL X2 with the default settings [36]. The same reference isolates were then used for phylogenetic analysis. For RNA segment 6, only the first 1,008 nucleotides were used; thus the HPR containing gaps and the remaining 3' end sequences were excluded from the analysis [7]. Phylogenetic trees were generated. Bootstrapping values (1000 replicates) were calculated. Branches with bootstrapping values ≥ 70% were considered significant, corresponding to a confidence interval ≥ 95% [37]. For visualization and printing of the trees, the NJPLOT program, Version 2.1 (Written by M. Gouy) was used.

### Divergence time estimation in a rooted phylogenetic tree

The computer program, BACKTRACK [3], which reads a phylogenetic tree with evolutionary distances and years of isolation for all the sequences and then generates a time interval for each inner node, was used to determine the timing of the evolutionary process among ISAV isolates.

## Competing interests

The authors declare that they have no competing interests.

## Authors' contributions

FSBK conceived the study, coordinated the research efforts, performed the sequence analysis, and drafted the manuscript. MGG made the veterinary investigations of the outbreaks, performed the necropsy and histological analysis, coordinated the laboratory investigation, and helped to write the manuscript. YW performed the phylogenetic analysis and helped to write the manuscript. MJTK performed the RT-PCR and sequence analysis of RNA segments 5 and 6 and edited the manuscript. VG and GL coordinated the laboratory investigations and shipping of samples to Canada, and helped to write the manuscript. SM and AL performed the RT-PCR and edited the manuscript. MH and FA coordinated the laboratory investigation and helped to draft the manuscript. ML and AG coordinated the sampling and field investigations and edited the manuscript. All co-authors read and approved the final manuscript.

## Supplementary Material

Additional file 1 **Origin and year of isolation of different ISAV isolates studied**. Table showing ISAV isolate name, country of origin, Year of isolation, and GenBank Accession numbers of Fusion and Haemagglutinin-Esterase genes. Click here for file

Additional file 2 **Phylogenetic tree of RNA segment 5 showing the relationships between all ISAV isolates**. For easy identification of the phylogenetic trees, some branches have been marked with letters or numbers; a letter or a number represents all the isolates in that branch. Click here for file

Additional file 3 **Phylogenetic tree of RNA segment 6 showing the relationships between all ISAV isolates**. For easy identification of the phylogenetic trees, some branches have been marked with letters or numbers; a letter or a number represents all the isolates in that branch. Click here for file

Additional file 4 **Source and characteristics of the new Chile ISAV strains**. Table showing characteristics of the 7 distinct Chile ISAV strains. Click here for file

Additional file 5 **Alignment of sequences in critical regions of the fusion glycoprotein of ISAV, updated from Kibenge *et al***. [3]** and Godoy *et al***. [4]. Comparison of amino acid sequences around the proteolytic cleavage site of the precursor F0 protein. The amino acid sequence corresponding to the fusion protein of the Chilean ISAV in this disease outbreak is highlighted in yellow. The designation of amino acid inserts IN1, IN2, and IN3 are as reported by Devold *et al*. [16]. The unique 11-amino acid insert found in the new Chilean ISAVs is designated IN4 [4]. Other sources of information are indicated as * Devold *et al*. [16], **Markussen *et al*. [6], and ***Plarre and Nylund (2004; SF83/04, GenBank Accession No. AY744392). Click here for file

Additional file 6 **Detailed analysis of the indel on segment 5 of ISAV**. Alignment of nucleotide sequences in the region with the insert mutation in segment 5 of ISAV. Click here for file

Additional file 7 **Alignment of sequences in critical regions of the haemagglutinin-esterase glycoprotein of ISAV, updated from Kibenge *et al***. [3]** and Godoy *et al***. [4]. Comparison of amino acid sequences in the highly polymorphic region (HPR) of the HE genes of various strains of ISAV. The amino acid sequence corresponding to the new Chile ISAVs is highlighted in yellow; only selected isolates representing the 24 HE-HPRs are listed. Outbreaks involving mixed HPRs are written in light blue. Sequences that are not determined are indicated by dots, and amino acid (aa) deletions are indicated by dashes. As previously reported [3], the figure also illustrates that deletion in the HPR of ≥ 13 amino acids (or if less, with deletion or mutation of the motif at amino acid positions ^352^FNT^354^), leads to pathogenicity and ability to replicate in cell culture with production of CPE and consequent virus isolation. ??? denotes no virus isolation; only HPR sequence was analyzed. Click here for file
