# Supplementary material for: Infectious salmon anaemia virus (ISAV) isolated from the ISA disease outbreaks in Chile diverged from ISAV isolates from Norway around 1996 and was disseminated around 2005, based on surface glycoprotein gene sequences
Source: Virol J. 2009 Jun 26;6:88. doi: 10.1186/1743-422X-6-88 (PMC2710322; doi:10.1186/1743-422X-6-88)
Supplement: Additional file 1 — Origin and year of isolation of different ISAV isolates studied. Table showing ISAV isolate name, country of origin, Year of isolation, and GenBank Accession numbers of Fusion and Haemagglutinin-Esterase genes. [file 1743-422X-6-88-S1.doc]

**Additional file 1.** Origin and year of isolation of different ISAV isolates studied

| **ISAV isolate and country of origin** | **GenBank Accession number** | | **Year of isolation** |
| --- | --- | --- | --- |
|  | RNA Segment 5  (Fusion protein) | RNA Segment 6  (Haemagglutinin-  esterase protein) |  |
| **CH01/08, Chile** | EU851042 | EU851043 | 2008 |
| **31648-3GH, Chile** | FJ592168 | FJ594334 | 2008 |
| **31588-14, Chile** | -^a^ | FJ594333 | 2008 |
| **33004-21, Chile** | - | FJ594332 | 2008 |
| **26572-6, Chile** | EU449765 | EU271682 | 2007 |
| **31591-6, Chile** | FJ592167 | FJ594331 | 2008 |
| **31587-9, Chile** | - | FJ594330 | 2008 |
| **31590-18, Chile** | FJ592166 | FJ594329 | 2008 |
| **31587-8, Chile** | FJ592165 | FJ594328 | 2008 |
| **31685-1, Chile** | FJ592164 | FJ594327 | 2008 |
| **PM-4165/8, Chile** | FJ592163 | FJ594326 | 2008 |
| **32719-108, Chile** | - | FJ594325 | 2008 |
| **31649-9, Chile** | - | FJ594324 | 2008 |
| **32913-66, Chile** | FJ592162^b^ | FJ594323 | 2008 |
| **32232-2032, Chile** | FJ592161 | FJ594322 | 2008 |
| **31905-7Cz, Chile** | FJ592160 | FJ594321 | 2008 |
| **1508-7, Chile** | EU849007 | EU849013 | 2008 |
| **31591-7, Chile** | FJ592159 | FJ594320 | 2008 |
| **33064-107, Chile** | - | FJ594319 | 2008 |
| **31648-5GH, Chile** | FJ592158 | FJ594318 | 2008 |
| **31589-17, Chile** | - | FJ594317 | 2008 |
| **33003-4, Chile** | - | FJ594316 | 2008 |
| **32916-1, Chile** | - | FJ594315 | 2008 |
| **32232-2044, Chile** | FJ592157 | FJ594314 | 2008 |
| **31905-9Cz, Chile** | FJ592156 | FJ594313 | 2008 |
| **31590-20, Chile** | FJ592155 | FJ594312 | 2008 |
| **31647-3GH, Chile** | FJ592154 | FJ594311 | 2008 |
| **31685-3, Chile** | FJ592153 | FJ594310 | 2008 |
| **33059-2, Chile** | - | FJ594309 | 2008 |
| **31647-8GH, Chile** | FJ592152 | FJ594308 | 2008 |
| **31667-5GH, Chile** | FJ592151 | FJ594307 | 2008 |
| **30290-2, Chile** | EU849010 | EU849017 | 2008 |
| **30741-8, Chile** | FJ592150 | FJ594306 | 2008 |
| **31667-3GH, Chile** | FJ592149 | FJ594305 | 2008 |
| **31790-3GH, Chile** | FJ592148 | FJ594304 | 2008 |
| **29560-2H, Chile** | - | FJ594303 | 2008 |
| **1508-6, Chile** | EU849005 | EU849012 | 2008 |
| **31790-9GH, Chile** | FJ592147 | FJ594302 | 2008 |
| **32089-P1, Chile** | FJ592146 | FJ594301 | 2008 |
| **31589-16, Chile** | - | FJ594300 | 2008 |
| **30290-5, Chile** | EU849011 | EU849018 | 2008 |
| **31689-1, Chile** | FJ592144 | FJ594299 | 2008 |
| **31689-4, Chile** | FJ592145 | FJ594298 | 2008 |
| **30740-3, Chile** | FJ592143 | FJ594297 | 2008 |
| **32325-4, Chile** | - | FJ594296 | 2008 |
| **26955-5, Chile** | - | EU625681 | 2007 |
| **27615-5, Chile** | - | EU625672 | 2008 |
| **26416-5, Chile** | - | EU849014 | 2007 |
| **26415-3, Chile** | EU449768^b^ | - | 2007 |
| **26560-10c, Chile** | - | EU625668 | 2007 |
| **26955-1, Chile** | - | EU625678 | 2007 |
| **26936-1c, Chile** | - | EU625677 | 2007 |
| **26416-6, Chile** | - | EU625674 | 2007 |
| **26955-3, Chile** | - | EU625679 | 2007 |
| **26955-4, Chile** | - | EU625680 | 2007 |
| **26560-4, Chile** | - | EU849015 | 2007 |
| **26936-1t, Chile** | EU849008 | EU625667 | 2007 |
| **PM-4165/11, Chile** | FJ592142 | FJ594295 | 2008 |
| **32980-5, Chile** | - | FJ594294 | 2008 |
| **31682-5, Chile** | FJ592141 | FJ594293 | 2008 |
| **30942/943, Chile** | FJ592140 | FJ594292 | 2008 |
| **26905-10, Chile** | EU849006 | EU625676 | 2007 |
| **26830, Chile** | EU449767 | EU625671 | 2007 |
| **26661, Chile** | - | EU625675 | 2007 |
| **31606-L, Chile** | - | FJ594291 | 2008 |
| **31592-2, Chile** | FJ592139 | FJ594290 | 2008 |
| **31592-4, Chile** | FJ592138 | FJ594289 | 2008 |
| **26936-2, Chile** | EU849009 | EU625673 | 2007 |
| **26829-2, Chile** | EU449766 | EU625670 | 2007 |
| **31686-2, Chile** | - | FJ594288 | 2008 |
| **31687-5, Chile** | FJ592137 | FJ594287 | 2008 |
| **31687-3, Chile** | FJ592136 | FJ594286 | 2008 |
| **33004-2, Chile** | - | FJ594285 | 2008 |
| **31606-H, Chile** | FJ592135 | FJ594282 | 2008 |
| **2006B13364, Chile** | FJ592134 | FJ594284 | 2008 |
| **26905-1c, Chile** | - | EU849016 | 2007 |
| **26905-1t, Chile** | EU552491 | EU625669 | 2007 |
| **31682-10, Chile** | FJ592133 | FJ594283 | 2008 |
| **U24636 (NO/1720/07)^c^, Chile** | EU130923 | AM941715 | 2007 |
| **26560-10t, Chile** | - | EU625666 | 2007 |
| **N32/98, Norway** | AY853921 | AF364883 | 1998 |
| **H97/04, Norway** | - | DQ108604 | 2004 |
| **SF83/04, Norway** | AY744392 | AY973190 | 2004 |
| **SK779/06, Norway** | EU118819 | EU118820 | 2006 |
| **ST25/97, Norway** | AY853926 | AF364885 | 1997 |
| **ST27/97, Norway** | AY853929 | AF364897 | 1997 |
| **ST28/97, Norway** | AY853927 | AF364875 | 1997 |
| **T90/04, Norway** | - | AY971666 | 2004 |
| **NT84/04, Norway** | - | AY973185 | 2004 |
| **97/09/615 (ISAV8), Norway** | DQ785238 | DQ785252 | 1997 |
| **MR104/05, Norway** | - | DQ108607 | 2005 |
| **93/09/2163 (ISAV9), Norway** | DQ785239 | DQ785253 | 1993 |
| **ISAV2/89, Norway** | DQ785232 | DQ785246 | 1989 |
| **H1/87, Norway** | AY853942 | AF364893 | 1987 |
| **5H07/92 (7/92), Norway** | AY853940 | AF364898 | 1992 |
| **N09/93, Norway** | AY853967 | AF364895 | 1993 |
| **390/98, Scotland, UK** | AF429988 | AF283997 | 1998 |
| **MR60/01, Norway** | AY853944 | AY127876 | 2001 |
| **H36/98, Norway** | AY853958 | AF302799 | 1998 |
| **810/9/99, Norway** | EF217313 | AF378180 | 1999 |
| **H56/00, Norway** | AY853946 | AF364880 | 2000 |
| **96/09/768 (ISAV5), Norway** | DQ785235 | DQ785249 | 1996 |
| **17/96, Norway** | - | AF364891 | 1996 |
| **96/09/734 (ISAV6), Norway** | DQ785236 | DQ785250 | 1996 |
| **SF18/96, Norway** | AY853963 | AF364869 | 1996 |
| **MR46/99, Norway** | AY853962 | AF364896 | 1999 |
| **H2143/89 (ISAV3), Norway** | DQ785233 | DQ785247 | 1989 |
| **N5/89, Norway** | - | AY127882 | 1989 |
| **SF48/99, Norway** | AY853966 | AF364878 | 1999 |
| **T33/98, Norway** | AY853969 | AF364887 | 1998 |
| **T22/96, Norway** | AY853968 | AF364889 | 1996 |
| **SF47/99, Norway** | AY853965 | AF364888 | 1999 |
| **96/09/1712 (ISAV1), Norway** | DQ785231 | DQ785245 | 2006 |
| **96/09/1729 (ISAV7), Norway** | DQ785237 | DQ785251 | 2006 |
| **5F72/02, Denmark, Faroes Islands** | AY853917 | - | 2002 |
| **F72d/02, Denmark, Faroes Islands** | - | AY971657 | 2002 |
| **F72b/02, Denmark, Faroes Islands** | - | AY971656 | 2002 |
| **MR14/95, Norway** | AY853925 | AF364873 | 1995 |
| **ISAV10/95, Norway** | DQ785240 | DQ785254 | 1995 |
| **Glesvaer, Norway** | - | AF220607 | 1990 |
| **90/09/400 (ISAV4), Norway** | DQ785234 | DQ785248 | 1990 |
| **6/91, Norway** | - | AF364894 | 1991 |
| **1490/98, Scotland, UK** | - | AF391126 | 1998 |
| **SF63/01, Norway** | - | AY127879 | 2001 |
| **SF57/00, Norway** | AY853939 | AF364890 | 2000 |
| **MR62/01, Norway** | AY853937 | AY127878 | 2001 |
| **54/00, Norway** | - | AF364884 | 2000 |
| **SF70/02, Norway** | AY853938 | AY127880 | 2002 |
| **5H02/89, Norway** | AY853930 | - | 1989 |
| **MR61/01, Norway** | AY853935 | AY127877 | 2001 |
| **MR71/02, Norway** | AY853936 | AY127881 | 2002 |
| **93/09/2264 (ISAV11), Norway** | DQ785241 | DQ785255 | 1993 |
| **N75/03, Norway** | AY853964 | AY971661 | 2003 |
| **T10/93, Norway** | AY853922 | AF302801 | 1993 |
| **SK-05/90 (200501B), Norway** | FJ594337^b^ | FJ594336 | 2005 |
| **MR102/05, Norway** | EU851044 | DQ108605 | 2005 |
| **SK-05/144 (200505A), Norway** | FJ594338 | FM203274 | 2005 |
| **MR105/05, Norway** | - | DQ108608 | 2005 |
| **MR103/05, Norway** | - | DQ108606 | 2005 |
| **ST21/96, Norway** | AY853952 | AF364886 | 1996 |
| **N49/99, Norway** | AY853950 | AF364876 | 1999 |
| **MR52/00, Norway** | AY853949 | AF364892 | 2000 |
| **ST30/97, Norway** | AY853953 | AY127875 | 1997 |
| **485/9/97, Norway** | EF217315 | AF378181 | 1997 |
| **ST26/97, Norway** | AY853951 | AF364879 | 1997 |
| **Vedoy/99, Norway** | - | AF302803 | 1999 |
| **NT92B/04, Norway** | - | AY973188 | 2004 |
| **NT87/04, Norway** | - | AY973186 | 2004 |
| **RPC/NB 04-085-1, Canada** | EF432567 | AY963263 | 2004 |
| **NS2003, Canada** | AY853919 | AY973182 | 2003 |
| **U5575-1, Canada** | EF217314 | AF294881 | 2000 |
| **NBISA01, Canada** | DQ465044 | AF283996 | 1998 |
| **RPC/NB 98-0280-2, Canada** | DQ440506 | AF294870 | 1998 |
| **7833-1, Chile** | DQ465046 | AF294879 | 1999 |
| **RPC/NB 01-0593-1, Canada** | DQ440508 | AY062033 | 2001 |
| **RPC/NB 01-0973-3, Canada** | DQ465047 | AY963266 | 2001 |
| **RPC/NB 02-1179-4, Canada** | DQ465045 | AY963264 | 2002 |
| **RPC/NB 02-0775-14, Canada** | DQ440507 | AY963265 | 2002 |
| **RPC/NB 98-049-1, Canada** | DQ465043 | AF294876 | 1998 |

^a^- denotes that a sequence is not available.

^b^denotes partial sequence.
