# Supplementary material for: Infectious salmon anaemia virus (ISAV) isolated from the ISA disease outbreaks in Chile diverged from ISAV isolates from Norway around 1996 and was disseminated around 2005, based on surface glycoprotein gene sequences
Source: Virol J. 2009 Jun 26;6:88. doi: 10.1186/1743-422X-6-88 (PMC2710322; doi:10.1186/1743-422X-6-88)
Supplement: Additional file 3 — Phylogenetic tree of RNA segment 6 showing the relationships between all ISAV isolates. For easy identification of the phylogenetic trees, some branches have been marked with letters or numbers; a letter or a number represents all the isolates in that branch. [file 1743-422X-6-88-S3.doc]

**Additional file 3**
