# Supplementary material for: Infectious salmon anaemia virus (ISAV) isolated from the ISA disease outbreaks in Chile diverged from ISAV isolates from Norway around 1996 and was disseminated around 2005, based on surface glycoprotein gene sequences
Source: Virol J. 2009 Jun 26;6:88. doi: 10.1186/1743-422X-6-88 (PMC2710322; doi:10.1186/1743-422X-6-88)
Supplement: Additional file 4 — Source and characteristics of the new Chile ISAV strains. Table showing characteristics of the 7 distinct Chile ISAV strains. [file 1743-422X-6-88-S4.doc]

**Additional file 4. Source and characteristics of the new Chile ISAV strains**

| **ISAV Clade** | **Isolate^1^** | **Fish company^2^** | **Sampling date** | **Segment 5**  **(11-aa insert)^3^** | **Segment 6 (HPR group)^4^** | **Clinical history** |
| --- | --- | --- | --- | --- | --- | --- |
| 2.2.2.1.2.1 | 31682-10 | 6 | July 2008 | No insert | HPR1c | 2007 Year Class; history of SRS^5^; ISA^6^ 43 weeks after transfer to sea |
|  | 31592-2 | 3 | July 2008 | No insert | New HPR  (12-aa deletion) | Diseased fish |
|  | 31606H | 11 | July 2008 | No insert | HPR1c | 2007 Year Class; history of SRS & Vibrio; ISA 3 weeks after transfer to sea |
|  | 31682-5 | 6 | July 2008 | No insert | HPR1c | 2007 Year Class; history of SRS; ISA 43 weeks after transfer to sea |
|  | 26936-2 | 5 | Nov 2007 | No insert | HPR5 | 2006 Year Class; history of amoeba disease; sampled 33 weeks after transfer to sea |
|  | 26936-1t | 5 | Nov 2007 | No insert | HPR5 |  |
|  | 2006B13364 | 14 | 2008 | No insert | HPR9 | Broodstock fish without clinical signs |
| 2.2.2.1.2.2 | 31687-5 | 11 | Aug 2008 | 11-aa insert | HPR7b | 2006 Year Class; ISA 50 weeks after transfer to sea; cage was split prior to disease outbreak |
|  | 31687-3 | 11 | Aug 2008 | 11-aa insert | HPR7b |  |
| 2.2.2.1.2.3 | 30290-5 | 6 | May 2008 | 11-aa insert | HPR7b | ISA |
|  | 30740-3 | 6 | June 2008 | 11-aa insert | HPR7b | 2006 Year Class; ISA 43 weeks after transfer to sea |
|  | 31689-1 | 6 | Aug 2008 | 11-aa insert | HPR7b | ISA |
|  | 32089-P1 | 12 | Aug 2008 | 11-aa insert | HPR7b | 2006 Year Class; Atlantic salmon jaundice & ISA 30 weeks after transfer to sea |
|  | 31689-4 | 6 | Aug 2008 | 11-aa insert | HPR7b | ISA |
|  | 1508-6 | 9 | Unknown | 11-aa insert | HPR7b | 2006 Year Class; history of SRS; ISA 14 weeks after transfer to sea |
|  | 1508-7 | 9 | Unknown | 11-aa insert | HPR7f |  |
| 2.2.2.1.2.4 | 31667-3GH | 6 | July 2008 | 11-aa insert | HPR7b | ISA |
|  | 31667-5GH | 6 | July 2008 | 11-aa insert | HPR7f |  |
|  | 30741-8 | 6 | June 2008 | 11-aa insert | HPR7b | ISA |
|  | 30290-2 | 6 | May 2008 | 11-aa insert | HPR7b | 2006 Year Class; history of SRS; ISA 39 weeks after transfer to sea |
| 2.2.2.1.2.5 | 31648-5GH | 10 | July 2008 | 11-aa insert | HPR7b | 2006 Year Class; ISA 55 weeks after transfer to sea |
|  | 31647-8GH | 10 | July 2008 | 11-aa insert | HPR7b | 2006 Year Class; ISA 78 weeks after transfer to sea |
|  | 31648-3GH | 10 | July 2008 | 11-aa insert | HPR7b | 2006 Year Class; ISA 55 weeks after transfer to sea |
|  | PM-4165/8 | 1 | - | 11-aa insert | HPR7b | ISA |
|  | 31905-7Cz | 10 | Aug 2008 | 11-aa insert | HPR7b | 2006 Year Class; ISA 62 weeks after transfer to sea |
|  | 31905-9Cz | 10 | Aug 2008 | 11-aa insert | HPR7b |  |
|  | PM-4165/11 | 1 | - | 11-aa insert | HPR7b | ISA |
|  | 31790-3GH | 6 | Aug 2008 | 11-aa insert | HPR7b | ISA |
|  | 31790-9GH | 6 | Aug 2008 | 11-aa insert | HPR7b |  |
|  | 31587-8 | 8 | July 2008 | 11-aa insert | HPR7b | 2005-2006 Year Class; ISA 90 weeks after transfer to sea |
|  | 30942/943 | 2 | June 2008 | 11-aa insert | HPR7b | ISA |
| 2.2.2.1.2.6 | 32232-2044 | 7 | July 2008 | 11-aa insert | HPR7b | 2006 Year Class; ISA 72 weeks after transfer to sea |
|  | 32232-2032 | 7 | July 2008 | 11-aa insert | HPR7b |  |
|  | 31591-6 | 13 | July 2008 | 11-aa insert | HPR7b | 2006 Year Class; ISA 71 weeks after transfer to sea |
|  | 31590-20 | 4 | July 2008 | 11-aa insert | HPR7b | 2007 Year Class; history of SRS; ISA 45 weeks after transfer to sea |
|  | 31647-3GH | 10 | July 2008 | 11-aa insert | HPR7b | 2006 Year Class; ISA 78 weeks after transfer to sea |
|  | 31685-1 | 13 | Aug 2008 | 11-aa insert | HPR7b | ISA |
|  | 31685-3 | 13 | Aug 2008 | 11-aa insert | HPR7b |  |
|  | 26572-6 | 2 | Oct 2007 | 11-aa insert | HPR7b | 2007 Year Class; history of SRS; ISA 19 weeks after transfer to sea |
|  | 31591-7 | 13 | July 2008 | 11-aa insert | HPR7b | 2006 Year Class; ISA 71 weeks after transfer to sea |
|  | 31590-18 | 4 | July 2008 | 11-aa insert | HPR7b | 2007 Year Class; history of SRS; ISA 45 weeks after transfer to sea |
| 2.2.2.1.2.7 | 26905-1t | 2 | Nov 2007 | 11-aa insert | HPR7b | 2006 Year Class; ISA 50 weeks after transfer to sea |
|  | 26905-10 | 2 | Nov 2007 | 11-aa insert | HPR2 & HPR7b |  |
|  | 26829-2 | 2 | Nov 2007 | 11-aa insert | HPR7b | 2007 Year Class; ISA 17 weeks after transfer to sea |
|  | U24636 | 2 | June 2007 | 11-aa insert | HPR7b | 2006 Year Class; history of SRS; original ISA outbreak in Chile, 63 weeks after transfer to sea |
|  | 26830 | 2 | Nov 2007 | 11-aa insert | HPR7b | 2007 Year Class; ISA 17 weeks after transfer to sea |

^1^Isolate name used is Case number of submitting diagnostic laboratory.

^2^Fish companies are numbered 1-14.

^3^Segment 5 11-amino acid insert from segment 2 (see also Supplementary Fig.2A).

^4^Segment 6 HPR group (see also Table 3 and Supplementary Fig.2B).

^5^SRS is abbreviation for Salmonid Rickettsial Septicaemia caused by *Piscirickettsia salmonis*.

^6^ISA is abbreviation for infectious salmon anaemia.
