# Supplementary material for: Infectious salmon anaemia virus (ISAV) isolated from the ISA disease outbreaks in Chile diverged from ISAV isolates from Norway around 1996 and was disseminated around 2005, based on surface glycoprotein gene sequences
Source: Virol J. 2009 Jun 26;6:88. doi: 10.1186/1743-422X-6-88 (PMC2710322; doi:10.1186/1743-422X-6-88)
Supplement: Additional file 5 — Alignment of sequences in critical regions of the fusion glycoprotein of ISAV, updated from Kibenge et al. [3] and Godoy et al. [4]. Comparison of amino acid sequences around the proteolytic cleavage site of the precursor F0 protein. The amino acid sequence corresponding to the fusion protein of the Chilean ISAV in this disease outbreak is highlighted in yellow. The designation of amino acid inserts IN1, IN2, and IN3 are as reported by Devold et al. [16]. The unique 11-amino acid insert found in the new Chilean ISAVs is designated IN4 [4]. Other sources of information are indicated as * Devold et al. [16], **Markussen et al. [6], and ***Plarre and Nylund (2004; SF83/04, GenBank Accession No. AY744392). [file 1743-422X-6-88-S5.doc]

**Additional file 5:**

**Alignment of Fusion protein amino acid sequences in trypsin cleavage area**

**ISAV isolate (GenBank Acc. No.) Predicted amino acid sequence aa inserted at cleavage site**

**& *in-vivo*/*in-vitro* virulence**

**250 260 270 280 290 300 310 320 330**

**Norway MR60/01 (AY853944) RANLANQHGWSKYSFGHSVHKLSN-----------QRA-----------FPGEEFIKCCGFTLGIGGAWFQAYLNGEVQGD 8 (IN1)***

**Norway MR46/99 (AY853962) RANLANQHGWSKYSF--------N-----------QRAPGGVLLTETITFPGEEFIKCCGFTLGIGGAWFQAYLNGEVQGD 11 (IN2)***

**Norway H2143/89(DQ785233) RASLANQHGWSKYSF--------N-----------QRAIPRTGYVRSA-FPGEEFIKCCGFTLGIGGAWFQAYLNGEVQGD 10 (IN3)***

**Norway SF70/02 (AY853938) RASLANQHGWSKYSF--------N-----------QRAIPRTGYVRSA-FPGEEFIKCCGFTLGIGGAWFQAYLNGEVQGD 10 (IN3)***

**Norway SF57/00 (AY853939) RASLANQHGWSKYSF--------N-----------QRAIPRTGYVRSA-FPGEEFIKCCGFTLGIGGAWFQAYLNGEVQGD 10 (IN3)***

**Norway MR62/01 (AY853937) RASLANQHGWSKYSF--------N-----------QRAIPRTGYVRSA-FPGEEFIKCCGFTLGIGGAWFQAYLNGEVQGD 10 (IN3)***

**Norway SF71/02 (AY853936) RASLANQHGWSKYSF--------N-----------HRAIPRTGYVRSA-FPGEEFIKCCGFTLGIGGAWFQAYLNGEVQGD 10 (IN3)***

**Norway MR61/01 (AY853935) RASLANQHGWSKYSF--------N-----------HRAIPRTGYVRSA-FPGEEFIKCCGFTLGIGGAWFQAYLNGEVQGD 10 (IN3)***

**Chile 1508-6 (EU849005) RAGLANQHGWSKYNF--------NKGKSANDIISDQRA-----------FPGEEFIKCCGFTLGIGGAWFQAYLNGEVQGD 11 (IN4)**

**Chile 1508-7 (EU849007) RAGLANQHGWSKYNF--------NKGKSANDIISDQRA-----------FPGEEFIKCCGFTLGIGGAWFQAYLNGEVQGD 11 (IN4)**

**Chile U24636 (EU130923) RAGLANQHGWSKYNF--------NKGKSANDIISDQRA-----------FPGEEFIKCCGFTLGIGGAWFQAYLNGEVQGD 11 (IN4)**

**Chile 26415-3 (EU449768) RAGLANQHGWSKYNF--------NKGKSANDIISDQRA-----------FPGEEFIKCCGFTLGIGGAWFQAYLNGEVQGD 11 (IN4)**

**Chile 26572-6 (EU449765) RAGLANQHGWSKYNF--------NKGKSANDIISDQRA-----------FPGEEFIKCCGFTLGIGGAWFQAYLNGEVQGD 11 (IN4)**

**Chile 26829-2 (EU449766) RAGLANQHGWSKYSF--------NKGKSANDIISDQRA-----------FPGEEFIKCCGFTLGIGGAWFQAYLNGEVQGD 11 (IN4)**

**Chile 26830 (EU449767) RAGLANQHGWSKYNF--------NKGKSANDIISDQRA-----------FPGEEFIKCCGFTLGIGGAWFQAYLNGEVQGD 11 (IN4)**

**Chile 26905-1 (EU552491) RAGLANQHGWSKYNF--------NKGKSANDIISDQRA-----------FPGEEFIKCCGFTLGIGGAWFQAYLNGEVQGD 11 (IN4)**

**Chile 26905-10 (EU849006) RAGLANQHGWSKYNF--------NKGKSANDIISDQRA-----------FPGEEFIKCCGFTLGIGGAWFQAYLNGEVQGD 11 (IN4)**

**Chile 30290-2 (EU849010) RAGLANQHGWSKYNF--------NKGKSANDIISDQRA-----------FPGEEFIKCCGFTLGIGGAWFQAYLNGEVQGD 11 (IN4)**

**Chile 30290-5 (EU849011) RAGLANQHGWSKYNF--------NKGKSANDIISDQRA-----------FPGEEFIKCCGFTLGIGGAWFQAYLNGEVQGD 11 (IN4)**

**Chile 30740-3 (FJ592143) RAGLANQHGWSKYNF--------NKGKSANDIISDQRA-----------FPGEEFIKCCGFTLGIGGAWFQAYLNGEVQGD 11 (IN4)**

**Chile 30741-8 (FJ592150) RAGLANQHGWSKYNF--------NKGKSANDIISDQRA-----------FPGEEFIKCCGFTLGIGGAWFQAYLNGEVQGD 11 (IN4)**

**Chile 30942/30943 (FJ592140) RAGLANQHGWSKYNF--------NKGKSANDIISDQRA-----------FPGEEFIKCCGFTLGIGGAWFQAYLNGEVQGD 11 (IN4)**

**Chile 31587-8 (FJ592165) RAGLANQHGWSKYNF--------NKGKSANDIISDQRA-----------FPGEEFIKCCGFTLGIGGAWFQAYLNGEVQGD 11 (IN4)**

**Chile 31589-17 (FJ786967) RAGLANQHGWSKYNF--------NKGKSANDIISDQRA-----------FPGEEFIKCCGFTLGIGGAWFQAYLNGEVQGD 11 (IN4)**

**Chile 31590-18 (FJ592166) RAGLANQHGWSKYNF--------NKGKSANDIISDQRA-----------FPGEEFIKCCGFTLGIGGAWFQAYLNGEVQGD 11 (IN4)**

**Chile 31590-20 (FJ592155) RAGLANQHGWSKYNF--------NKGKSANDIISDQRA-----------FPGEEFIKCCGFTLGIGGAWFQAYLNGEVQGD 11 (IN4)**

**Chile 31591-6 (FJ592167) RAGLANQHGWSKYNF--------NKGKSANDIISDQRA-----------FPGEEFIKCCGFTLGIGGAWFQAYLNGEVQGD 11 (IN4)**

**Chile 31591-7 (FJ592159) RAGLANQHGWSKYNF--------NKGKSANDIISDQRA-----------FPGEEFIKCCGFTLGIGGAWFQAYLNGEVQGD 11 (IN4)**

**Chile 31647-3GH (FJ592154) RAGLANQHGWSKYNF--------NKGKSANDIISDQRA-----------FPGEEFIKCCGFTLGIGGAWFQAYLNGEVQGD 11 (IN4)**

**Chile 31647-8GH (FJ592152) RAGLANQHGWSKYNF--------NKGKSANDIISDQRA-----------FPGEEFIKCCGFTLGIGGAWFQAYLNGEVQGD 11 (IN4)**

**Chile 31648-3GH (FJ592168) RAGLANQHGWSKYNF--------NKGKSANDIISDQRA-----------FPGEEFIKCCGFTLGIGGAWFQAYLNGEVQGD 11 (IN4)**

**Chile 31648-5GH (FJ592158) RAGLANQHGWSKYNF--------NKGKSANDIISDQRA-----------FPGEEFIKCCGFTLGIGGAWFQAYLNGEVQGD 11 (IN4)**

**Chile 31667-3GH (FJ592149) RAGLANQHGWSKYNF--------NKGKSANDIISDQRA-----------FPGEEFIKCCGFTLGIGGAWFQAYLNGEVQGD 11 (IN4)**

**Chile 31667-5GH (FJ592151) RAGLANQHGWSKYNF--------NKGKSANDIISDQRA-----------FPGEEFIKCCGFTLGIGGAWFQAYLNGEVQGD 11 (IN4)**

**Chile 31685-1 (FJ592164) RAGLANQHGWSKYNF--------NKGKSANDIISDQRA-----------FPGEEFIKCCGFTLGIGGAWFQAYLNGEVQGD 11 (IN4)**

**Chile 31685-3 (FJ592153) RAGLANQHGWSKYNF--------NKGKSANDIISDQRA-----------FPGEEFIKCCGFTLGIGGAWFQAYLNGEVQGD 11 (IN4)**

**Chile 31687-3 (FJ592136) RAGLANQHGWSKYNF--------NKGKSANDIISDQRA-----------FPGEEFIKCCGFTLGIGGAWFQAYLNGEVQGD 11 (IN4)**

**Chile 31687-5 (FJ592137) RAGLANQHGWSKYNF--------NKGKSANDIISDQRA-----------FPGEEFIKCCGFTLGIGGAWFQAYLNGEVQGD 11 (IN4)**

**Chile 31689-1 (FJ592144) RAGLANQHGWSKYNF--------NKGKSANDIISDQRA-----------FPGEEFIKCCGFTLGIGGAWFQAYLNGEVQGD 11 (IN4)**

**Chile 31689-4 (FJ592145) RAGLANQHGWSKYNF--------NKGKSANDIISDQRA-----------FPGEEFIKCCGFTLGIGGAWFQAYLNGEVQGD 11 (IN4)**

**Chile 31790-3GH (FJ592148) RAGLANQHGWSKYNF--------NKGKSANDIISDQRA-----------FPGEEFIKCCGFTLGIGGAWFQAYLNGEVQGD 11 (IN4)**

**Chile 31790-9GH (FJ592147) RAGLANQHGWSKYNF--------NKGKSANDIISDQRA-----------FPGEEFIKCCGFTLGIGGAWFQAYLNGEVQGD 11 (IN4)**

**Chile 31905-7Cz (FJ592160) RAGLANQHGWSKYNF--------NKGKSANDIISDQRA-----------FPGEEFIKCCGFTLGIGGAWFQAYLNGEVQGD 11 (IN4)**

**Chile 31905-9Cz (FJ592156) RAGLANQHGWSKYNF--------NKGKSANDIISDQRA-----------FPGEEFIKCCGFTLGIGGAWFQAYLNGEVQGD 11 (IN4)**

**Chile 32089-P1 (FJ592146) RAGLANQHGWSKYNF--------NKGKSANDIISDQRA-----------FPGEEFIKCCGFTLGIGGAWFQAYLNGEVQGD 11 (IN4)**

**Chile 32232-2032LK (FJ592161) RAGLANQHGWSKYNF--------NKGKSANDIISDQRA-----------FPGEEFIKCCGFTLGIGGAWFQAYLNGEVQGD 11 (IN4)**

**Chile 32232-2044LK (FJ592157) RAGLANQHGWSKYNF--------NKGKSANDIISDQRA-----------FPGEEFIKCCGFTLGIGGAWFQAYLNGEVQGD 11 (IN4)**

**Chile PM-4165#8 (FJ592163) RAGLANQHGWSKYNF--------NKGKSANDIISDQRA-----------FPGEEFIKCCGFTLGIGGAWFQAYLNGEVQGD 11 (IN4)**

**Chile PM-4165#11 (FJ592142) RAGLANQHGWSKYNF--------NKGKSANDIISDQRA-----------FPGEEFIKCCGFTLGIGGAWFQAYLNGEVQGD 11 (IN4)**

**Norway SK779/06(EU118819) RAGLANQHGWSKYNF--------N-----------QRA-----------FPGEEFIKCCGFTLGIGGAWFQAYLNGEVQGD 0 (ISAV HPR0)****

**Additional file 5 continued:**

**Norway SF83/04 (AY744392) RAGLANQHGWSKYNF--------N-----------QRA-----------FPGEEFIKCCGFTLGIGGAWFQAYLNGEVQGD 0 (low virul. ISAV)*****

**Can RPC/NB 04-085-1 (EF432567) RANLANQHGWSKYSF--------Y-----------PRA-----------FPGEEFIKCCGFTLGIGGAWFQAYLNGEVQGD 0 (low virul. ISAV)**

**Norway 5ST27/97 (AY853929) RAGLANQHGWSKYNF--------N-----------LRA-----------FPGEEFIKCCGFTLGIGGAWFQAYLNGEVQGD 0**

**Norway SK-05:90 (FJ594337) RAGLANQHGWSKYNF--------N-----------LRA-----------FPGEEFIKCCGFTLGIGGAWFQAYLNGEVQGD 0**

**Norway SK-05:144 (FJ594338) RAGLANQHGWSKYNF--------N-----------LRA-----------FPGEEFIKCCGFTLGIGGAWFQAYLNGEVQGD 0**

**Chile 2006B-13364 (FJ592134) RAGLANQHGWSKYNF--------N-----------LRA-----------FPGEEFIKCCGFTLGIGGAWFQAYLNGEVQGD 0 (low virul. ISAV)**

**Chile 26936-1 (EU849008) RAGLANQHGWSKYNF--------N-----------LRA-----------FPGEEFIKCCGFTLGIGGAWFQAYLNGEVQGD 0**

**Chile 26936-2 (EU849009) RAGLANQHGWSKYNF--------N-----------LRA-----------FPGEEFIKCCGFTLGIGGAWFQAYLNGEVQGD 0**

**Chile 31592-4 (FJ592138) RAGLANQHGWSKYNF--------N-----------LRA-----------FPGEEFIKCCGFTLGIGGAWFQAYLNGEVQGD 0**

**Chile 31592-2 (FJ592139) RAGLANQHGWSKYNF--------N-----------LRA-----------FPGEEFIKCCGFTLGIGGAWFQAYLNGEVQGD 0**

**Chile 31606-H (FJ592135) RAGLANQHGWSKYNF--------N-----------LRA-----------FPGEEFIKCCGFTLGIGGAWFQAYLNGEVQGD 0**

**Chile 31682-5 (FJ592141) RAGLANQHGWSKYNF--------N-----------LRA-----------FPGEEFIKCCGFTLGIGGAWFQAYLNGEVQGD 0**

**Chile 31682-10 (FJ592133) RAGLANQHGWSKYNF--------N-----------LRA-----------FPGEEFIKCCGFTLGIGGAWFQAYLNGEVQGD 0**

**Norway SF14/95 (AY853925) RADLANQHGWSKYNF--------N-----------LRA-----------FPGEEFIKCCGFTLGIGGAWFQAYLNGEVQGD 0***

**Norway N32/98 (AY853921) RAGLANQHGWSKYNF--------N-----------LRA-----------FPGEEFIKCCGFTLGIGGAWFQAYLNGEVQGD 0***

**Can NovaScotia NS2003(AY853919) RANLANQHGWSKYSF--------N-----------LRA-----------FPGEEFIKCCGFTLGIGGAWFQAYLNGEVQGD 0**

**Norway 810/9/99 (EF217313) RANLANQHGWSKYSF--------N-----------LRA-----------FPGEEFIKCCGFTLGIGGAWFQAYLNGEVQGD 0 (high virul. ISAV)**

**Scotland 390/98(AF429988) RANLANQHGWSKYSF--------N-----------LRA-----------FPGEEFIKCCGFTLGIGGAWFQAYLNGEVQGD 0**

**Norway 485/9/97(EF217315) RANLANQHGWSKYSF--------N-----------LRA-----------FPGEEFIKCCGFTLGIGGAWFQAYLNGEVQGD 0**

**Can NovaScotiaU5575-1(EF217314) RANLANQHGWSKYSF--------N-----------LRA-----------FPGEEFIKCCGFTLGIGGAWFQAYLNGEVQGD 0**

**Can NBISA01(DQ465044) KANFVNKHGWSKYNF--------N-----------LRG-----------FPGEEFIKCCGFTLGVGGAWFQAYLNGMVQGD 0 (high virul. ISAV)**

**Can RPC/NB 98-049-1(DQ465043) KANFVNKHGWSKYNF--------N-----------LRG-----------FPGEEFIKCCGFTLGVGGAWFQAYLNGMVQGD 0 (high virul. ISAV)**

**Can RPC/NB 02-1179-4 (DQ465045) KANFVNKHGWSKYNF--------N-----------LRG-----------FPGEEFIKCCGFTLGVGGAWFQAYLNGMVQGD 0**

**Chile 7833-1(DQ465046) KANFVNKHGWSKYNF--------N-----------LRG-----------FPGEEFIKCCGFTLGVGGAWFQAYLNGMVQGD 0**

**Can RPC/NB 02-0775-14(DQ440507) KANFVNKHGWSKYNF--------N-----------LRG-----------FPGEEFIKCCGFTLGVGGAWFQAYLNGMVQGD 0**

**Can RPC/NB 01-0593-1(DQ440508) KANFVNKHGWSKYNF--------N-----------LRG-----------FPGEEFIKCCGFTLGVGGAWFQAYLNGMVQGD 0**

**Can RPC/NB 01-0973-3(DQ465047) KANFVNKHGWSKYNF--------N-----------LRG-----------FPGEEFIKCCGFTLGVGGAWFQAYLNGMVQGD 0**

**Can RPC/NB 98-0280-2(DQ440506) KANFVNKHGWSKYNF--------N-----------LRG-----------FPGEEFIKCCGFTLGVGGAWFQAYLNGMVQGD 0**
