# Supplementary material for: Infectious salmon anaemia virus (ISAV) isolated from the ISA disease outbreaks in Chile diverged from ISAV isolates from Norway around 1996 and was disseminated around 2005, based on surface glycoprotein gene sequences
Source: Virol J. 2009 Jun 26;6:88. doi: 10.1186/1743-422X-6-88 (PMC2710322; doi:10.1186/1743-422X-6-88)
Supplement: Additional file 6 — Detailed analysis of the indel on segment 5 of ISAV. Alignment of nucleotide sequences in the region with the insert mutation in segment 5 of ISAV. [file 1743-422X-6-88-S6.doc]

**Additional file 7:**

**Additional file 7 continued:**

**Additional file 7 continued:**
