# Supplementary material for: Infectious salmon anaemia virus (ISAV) isolated from the ISA disease outbreaks in Chile diverged from ISAV isolates from Norway around 1996 and was disseminated around 2005, based on surface glycoprotein gene sequences
Source: Virol J. 2009 Jun 26;6:88. doi: 10.1186/1743-422X-6-88 (PMC2710322; doi:10.1186/1743-422X-6-88)
Supplement: Additional file 7 — Alignment of sequences in critical regions of the haemagglutinin-esterase glycoprotein of ISAV, updated from Kibenge et al. [3] and Godoy et al. [4]. Comparison of amino acid sequences in the highly polymorphic region (HPR) of the HE genes of various strains of ISAV. The amino acid sequence corresponding to the new Chile ISAVs is highlighted in yellow; only selected isolates representing the 24 HE-HPRs are listed. Outbreaks involving mixed HPRs are written in light blue. Sequences that are not determined are indicated by dots, and amino acid (aa) deletions are indicated by dashes. As previously reported [3], the figure also illustrates that deletion in the HPR of ≥ 13 amino acids (or if less, with deletion or mutation of the motif at amino acid positions 352FNT354), leads to pathogenicity and ability to replicate in cell culture with production of CPE and consequent virus isolation. ??? denotes no virus isolation; only HPR sequence was analyzed. [file 1743-422X-6-88-S7.doc]

**Additional file 6:**

**Alignment of amino acid sequences in the highly polymorphic region (HPR) of the HE gene**

**HPR ISAV isolate(GenBank Acc. No.) Predicted amino acid sequence aa deleted in HPR Virus isolated?**

**Group**

**330 340 350 360 370**

**HPR0 Norway farmed salmonSK779/06 (EU118820) SKLQRNITDVKIRVDAIPPQLNQTFNTNQVEQPANSVLSNIFISMGVAGF 0 No**

**HPR0 Scotland wild salmon(AJ440971) SKLQRNITDVKIRVDAIPPQLNQTFNTNQVEQPANSVLSNIFISMGVAGF 0 No**

**HPR0 Can Nova Scotia farmed salmon(AY646058) ....RNITDVKIRVDAIPPQLNQTFNTNQVEQPATSVLSNIFISMGV... 0 No CPE in SHK**

**HPR0 Can NB farmed salmon(AY646060) ....RNITDVKIRVDAIPPQLNQTFNTNQVEQPATSVLSNIFISMGV... 0 No CPE in SHK**

**HPR0 USA Maine farmed salmon(AY973194) SKLQRNITDVKIRVDAIPPQLNQTFNTNQVEQPSTSVLSNIFISMGVAGF 0 No**

**HPR0 Chile 13492-9 (EU999161) SKLQRNITDVKIRVDAIPPQLNQTFNTNQVEQPATSVLSNIFISMGVAGF 0 No**

**PR12 Norway 97/09/393(AF427070) SKLQRNITDVKIRVDAI-------FNTNQVEQPATSVLSNIFISMGVAGF 7 No**

**HPR6 Norway ST25/97(AF364885) SKLQRNITDVKIRVDAI-----------QVEQPATSVLSNIFISMGVAGF 11 Yes**

**HPR14 Norway 485/9/97(AY378181) SKLQRNITDVKIRVDA-----------NQVEQPATSVLSNIFISMGVAGF 11 Yes**

**new HPR Chile 31592-4 (FJ594289) SKLQRNITD-----------LK-TFNTNQVEQPATSVLSNIFISMGVAGF 12 ???**

**PR16 Norway 94/09/579(AF427073) S-------------DA--------FNTNQVEQPATSVLSNIFISMGVAGF 12 (no ^333^NIT^335^) No**

**Can RPC/NB 04-085-1(AY963263) SKLQRNITDVKIRVDAIPPQLNQTFNTN-------------FISMGVAGF 13 Yes**

**Norway SK-05:90 (FJ594336) SKLQRNITDVKIR-------LE-------VEQPATSVLSNIFISMGVAGF 14 Yes**

**HPR12 Norway N5/89(AY127882) SKLQRNITDVKIRVDAIPPQLNQT---------------NIFISMGVAGF 15 Yes**

**HPR9b Chile 2006B-13364 ((FJ594284) SKLQRNITDVKIRVDAIPPQLNQTFNT---------------ISMGVAGF 15 Yes**

**HPR9 Norway 47/99 SF(AF364888) SKLQRNITDVKIRVDAIPPQLNQTFNT-----------------MGVAGF 17 Yes**

**HPR4 Norway T10/93(AF302801) SKLQRNITDVKIRVDAIPPQL-----------------SNIFISMGVAGF 17 Yes**

**HPR3 Can Nova Scotia U5575-1(AF294881) SKLQRNITDVKIRVDAIPPQLNQT-----------------FISMGVAGF 17 Yes**

**HPR3 Chile 29002 (FJ786968) SKLQRNITDVKIRVDAIPPQLNQT-----------------FISMGVAGF 17 ???**

**HPR3a Chile 32141 (FJ786969) SKLQRNITDVKIGVDAIPPRLNQT-----------------FISMGVAGF 17 ???**

**HPR4c Chile 26416-6 (FJ786970) SKLQRNITDVKIRVDAIPPQL------------------NIFISMGVAGF 18 Yes**

**HPR4c Chile 26560-10 (EU625666) SKLQRNITDVKIRVDAIPPQL------------------NIFISMGVAGF 18 Yes**

**HPR10 Norway MR52/00(AF364892) SKLQRNITDVKIK------------------QPATSVLSNIFISMGVAGF 18 Yes**

**HPR13 Faroe Islands 1173/01/12(AJ440970) SKLQRNITDVK-------------------EQPANSVLSNIFISMGVAGF 19 Yes**

**HPR11 Norway 54/00 SF(AF364884) SKLQRNITDVKIRVDAIPP-------------------RNIFISMGVAGF 19 Yes**

**HPR2 Scotland 1490/98(AF391126) SKLQRNITDVKIRVDAIPPQLNQT--------------------MGVAGF 20 Yes**

**HPR2 Chile 26905-10b (FJ786971) SKLQRNITDVKIRVDAIPPQLNQT--------------------MGVAGF 20 Yes**

**HPR2 Chile 27102-1 (FJ786972) SKLQRNITDVKIRVDAIPPQLNQT--------------------MGVAGF 20 ???**

**HPR2 Chile 30735-2 (FJ786973) SKLQRNITDVKIRVDAIPPQLNQT--------------------MGVAGF 20 ???**

**HPR2c Chile 30735-2c (FJ786974) SKLQRNITDVKIRVYAIPPQLNQT--------------------MGVAGF 20 ???**

**HPR2d Chile 29678-4 (FJ786975) SKLHRNITDVKIRVDAIPPQLNQTF--------------------GVAGF 20 ???**

**PR2 Can F1-97(AF427045) SKLQRNITDVKIRVDAIPPQLNQT-------------L-------GVAGF 20 Yes**

**Norway Glesvaer/2/90(AF283998) SKLQRNITDVK-------------TCNICVEQ----HL--I-ISMGVAGF 20 (FNT → TCN) Yes**

**HPR5 Norway MR14/95(AF364873) SKLQRNITDVKIRVDAIPPQL---------------------ISMGVAGF 21 Yes**

**HPR5 Chile 26936-1 (EU625667) SKLQRNITDVKIRVDAIPPQL---------------------ISMGVAGF 21 Yes**

**HPR1 Norway H1/87(AF364893) SKLQRNITDVK---------------------PATSVLSNIFISMGVAGF 21 Yes**

**HPR1b Chile 29944-4 (FJ786976) SKLQRNITDV----------------------WA-SVLSNIFISMGVAGF 23 ???**

**HPR1c Chile 31606-H (FJ594282) SKLQRNITDV-----------------------ATSVLSNIFISMGVAGF 23 ???**

**HPR7b Scotland 390/98(AF283997) SKLQRNITDVK-----------------------TSVLSNIFISMGVAGF 23 Yes**

**HPR7b Chile 13492-9 (EU999162) SKLQRNITDVK-----------------------TSVLSNIFISMGVAGF 23 ???**

**HPR7b Chile NO/1720/07 (AM941715) SKLQRNITDVK-----------------------TSVLSNIFISMGVAGF 23 Yes**

**HPR7b Chile 1508-6 (EU849012) SKLQRNFTDVK-----------------------TSVLSNIFISMGVAGF 23 ???**

**HPR7c Chile 24984-1 (FJ786977) SKLQRNITDVK-----------------------TSVLSNIFISTGVAGF 23 Yes**

**HPR7b Chile 26560-10b (FJ786978) SKLQRNITDVK-----------------------TSVLSNIFISMGVAGF 23 Yes**

**HPR7b Chile 26416-6 (EU625674) SKLQRNITDVK-----------------------TSVLSNIFISMGVAGF 23 Yes**

**HPR7b Chile 26905-10 (EU625676) SKLQRNITDVK-----------------------TSVLSNIFISMGVAGF 23 Yes**

**HPR7b Chile 31903-3Bz (FJ786982) SKLQRNITDVK-----------------------TSVLNNILIYMGVTFG 23 ???**

**HPR7e Chile 29681-2 (FJ786979) SKFQRNITDVK-----------------------TFVLSNIFISMGVAGF 23 ???**

**HPR7f Chile 1508-7 (EU849013) SKLQRNITDVK-----------------------TSVLSNTFIYMGVAGF 23 ???**

**HPR7g Chile 31807-2 (FJ786980) SKLQRNIPDVK-----------------------TYVLSNILIYMGVAGF 23 ???**

**HPR7h Chile 31903-3Br (FJ786981) SKLQRNITDVK-----------------------TSVLNNILIYMGVTFG 23 ???**

**HPR7i Chile 31991-3N (FJ786983) SKLQRNFTDVK-----------------------TSVLSNILISMGVAGF 23 ???**

**Additional file 6 continued:**

**HPR15 Norway 810/9/99(AF378180) SKLQRNITDV--------------------E---TSVLSNIFISMGVAGF 23 Yes**

**HPR15 Chile 8 (FJ786984) ......TTD--------------------VE---TSVLSNIFISMGVAGF 23 Yes**

**HPR15b Chile 31150-3 (FJ786985) SKLQRNITDV-----------L------------TSVLSNIFISMGVAGF 23 ???**

**HPR15c Chile 27767 (FJ786986) SKLQRNIT---------------------VEK--TSVLSNIFISMGVAGF 23 ???**

**HPR15d Chile 30408-6 (FJ786987) SHLQRNIT---------P-----------V---VTYVLSNIFISMGVAGF 24 ???**

**HPR15e Chile 32246 (FJ786988) SKFQRNITDV-----------------------TTSVLSNIFISMGVAGF 23 ???**

**HPR8 Norway 48/99 SF(AF364878) SKLQRNITDVKIRVDAIPPQL------------------------GVAGF 23 Yes**

**HPR20 Can RPC/NB 98-0280-2(AF294870) GKLGRNITDVNNRVDAI-------LGVNQVEQPSTSVPSNIFISMGVAGF 7 (FNT → LGV) Yes**

**HPR21 Can NBISA01(AF283996) GKLGRNITDVNNRVDAIPPQL-----------------SNIFISMGVAGF 17 Yes**

**HPR21 Chile 7833-1(AF294879) GKLGRNITDVNNRVDAIPPQL-----------------SNIFISMGVAGF 17 Yes**

**Can NB1330-2(AY646063) ....RNITDVNNRVDAIPPQLNQT--------------------MGV... 20 Yes**

**__ __________ __**

**HPR**
